# Supplementary material for: The interactome of intact mitochondria by cross-linking mass spectrometry provides evidence for coexisting respiratory supercomplexes
Source: Mol Cell Proteomics. 2017 Dec 8;17(2):216–32. doi: 10.1074/mcp.RA117.000470 (PMC5795388; doi:10.1074/mcp.RA117.000470)
Supplement: Supplemental Data [file supp_RA117.000470_133922_0_supp_23978_fzffwf.zip › spectra_annotation/mito_spectra_annotation/145-1-4-1-18-1.pdf]

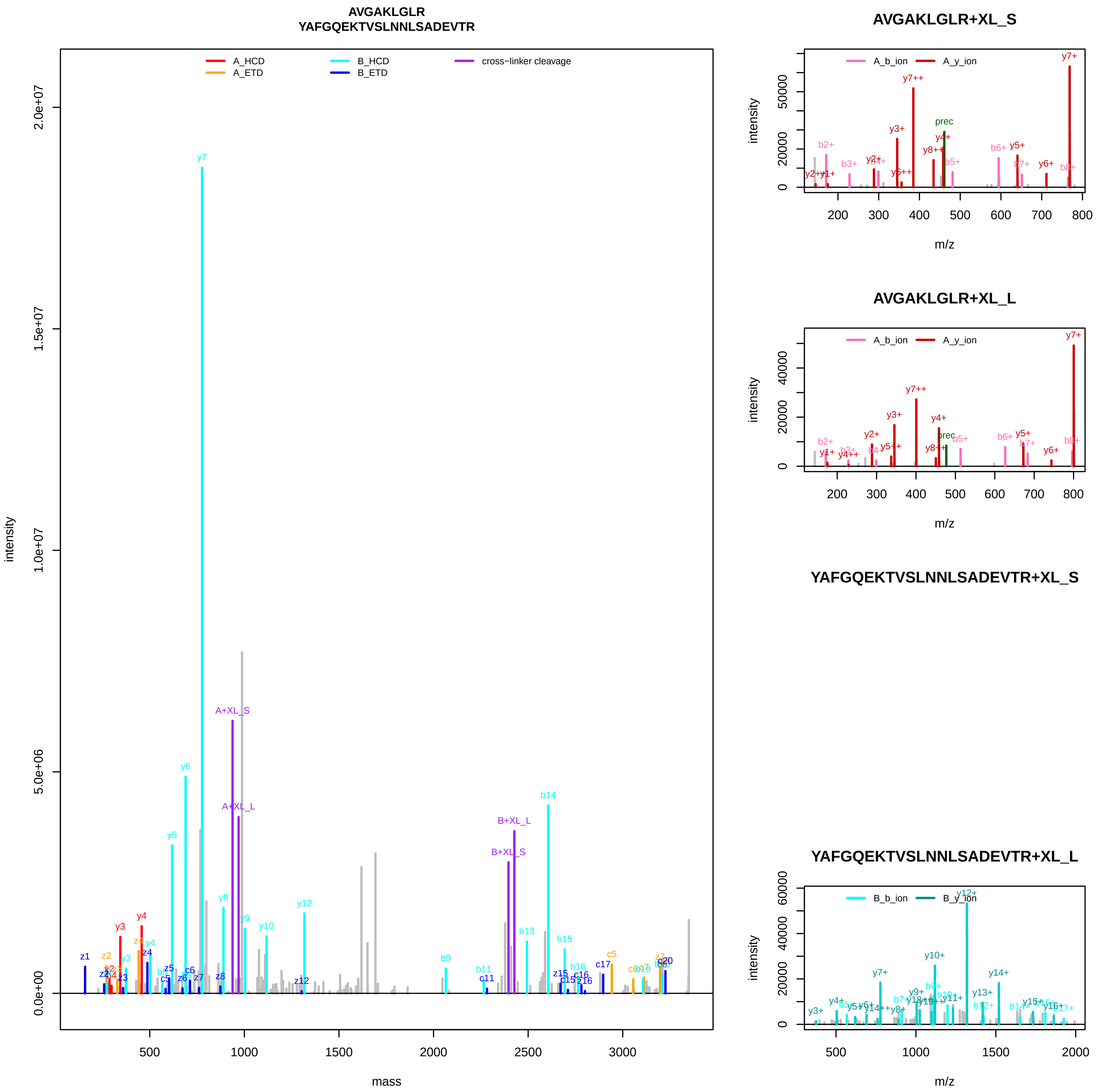
AVGAKLGLR
YAFGQEKTVSLNNLSADEVTR
A_HCD
A_ETD
B_HCD
B_ETD
cross−linker cleavage
intensity
mass
0.0e+00
5.0e+06
1.0e+07
1.5e+07
2.0e+07
500
1000
1500
2000
2500
3000
y7
y6
y5
y8
y9
y10
y12
y3
y4
A+XL_S
A+XL_L
B+XL_L
B+XL_S
b14
b13
b15
b16
b9
b11
c11
c5
c17
c20
z1
z2
z4
z5
z6
z7
z8
z12
z15
z16
c5
c6
c15
c16
AVGAKLGLR+XL_S
A_b_ion
A_y_ion
y7+
y7++
y3+
y4+
prec
y8++
y2+
b2+
b3+
b5+
b6+
y5+
b7+
y6+
b8+
y6++
y1+
m/z
intensity
AVGAKLGLR+XL_L
A_b_ion
A_y_ion
y7+
y7++
y3+
y4+
prec
b5+
b6+
y5+
b7+
y6+
b8+
b2+
y2+
y1+
y4++
y5++
y8++
m/z
intensity
YAFGQEKTVSLNNLSADEVTR+XL_S
YAFGQEKTVSLNNLSADEVTR+XL_L
B_b_ion
B_y_ion
y12+
y10+
y7+
y14+
y13+
y3+
y4+
b9+
b10+
y11+
m/z
intensity
